# The Safety Assessment of Toxic Metals in Commonly Used Herbs, Spices, Tea, and Coffee in Poland

**DOI:** 10.3390/ijerph18115779

**Published:** 2021-05-27

**Authors:** Grażyna Kowalska

**Affiliations:** Department of Tourism and Recreation, University of Life Sciences in Lublin, 15 Akademicka Street, 20-950 Lublin, Poland; grazyna.kowalska@up.lublin.pl; Tel.: +48-81-445-66-53

**Keywords:** contamination of heavy metals, herbs, spices, tea, coffee, risk assessment

## Abstract

The presented study was aimed at the determination of the level of contamination with heavy metals (Cd, Pb, As, and Hg) in 240 samples of plant materials, i.e., herbal raw materials, spices, tea, and coffee. Moreover, a probabilistic risk assessment (noncarcinogenic and carcinogenic risks) was estimated by models including target hazard quotient (THQ) and cancer risk (CR). The samples were subjected to microwave mineralisation with the use of HNO_3_ (65%), while the determination of the content of the elements was performed with the use of inductively coupled plasma mass spectrometer (ICP–MS) and a mercury analyser. The element which was characterised by the highest level of accumulation in the analysed samples was lead (from 0.010 to 5.680 mg/kg). Among the heavy metals under analysis, the lowest concentration was noted in the case of mercury (from 0.005 to 0.030 mg/kg). A notably higher level of contamination with heavy metals was noted in the analysed samples of herbs and spices (0.005–5.680 mg/kg), compared to samples of tea and coffee (0.005–0.791 mg/kg). According to the guidelines of the World Health Organisation (WHO) concerning the limits of contamination of samples of herbal raw materials with heavy metals, lead levels exceeding the limits were only noted in 24 samples of herbs (18%). In all of the analysed samples of spices, tea, and coffee, no instances of exceeded limits were noted for any of the analysed heavy metals. The values of TTHQmax (in relation to the consumption of the analysed products) were as follows: up to 4.23 × 10^−2^ for spices, up to 2.51 × 10^−1^ for herbs, up to 4.03 × 10^−2^ for China tea, and up to 1.25 × 10^−1^ for roasted coffee beans. As the value of THQ ≤1, there is no probability of the appearance of undesirable effects related to the consumption of the analysed group of raw materials and products of plant origin. The CR value for As (max. value) was 1.29 × 10^−5^, which is lower than the maximum acceptable level of 1 × 10^−4^ suggested by United States Environmental Protection Agency (USEPA).

## 1. Introduction

Spices and herbs grown in various regions of Poland are used as aromatic additives enhancing the sensory values of food. Although they do not contain nutrients, they are characterised by medicinal properties through antioxidant, antibacterial, anti-inflammatory, and anticarcinogenic effects. The therapeutic effect of herbal raw materials results from the medicinal properties of biologically active compounds included in their composition, such as essential oils, glycosides, vitamins, alkaloids, and tannins. Herbs and spices are also used for the purpose of alleviation of symptoms of stress, fatigue, unrest, or nervousness [1,2].

Brews of tea and coffee are the two most popular drinks. Numerous literature reports have taken note of the beneficial effect of moderate consumption of tea and coffee on human health through the regulation of the level of sugar in the blood, as well as prevention of diseases of the circulatory system and digestive system, cancers, and Parkinson’s and Alzheimer’s diseases [3,4,5].

However, in spite of the numerous beneficial characteristics of those commonly used raw materials and products of plant origin, they can also contain toxic substances accumulated from the environment. In addition, they can also be contaminated with heavy metals in the course of cultivation, as well as in the course of the processes involved in their storage and processing [6]. The accumulation of heavy metals in soil, water, and air results from incorrect waste management and from the rapid industrialisation and urbanisation of agricultural regions. Sources of inorganic contaminants of the air can be sought in the production of coal, petroleum oil, nonferrous metals, and cement. Soil contaminants are formed as a result of agricultural use of fertilisers and plant protection agents, including pesticides and fungicides containing mercury and arsenic. The water environment is contaminated with heavy metals as a result of human activity related to wastewater treatment and drying, as well as with the disposal of sewage and sanitary wastes from households [7,8]. Toxic elements are widely proliferated in the environment and enter the food chain, appearing in various concentrations in food and feeds. The absorption and bioaccumulation of those substances have a negative effect on the health of consumers. The most toxic elements are heavy metals, such as Cd—cadmium, Pb—lead, As—arsenic, and Hg—mercury, which can cause a broad array of toxic and mutagenic effects. They disturb the ionic balance and mineral regulation, stimulate oxidative damage to cellular structures and DNA, and cause neoplastic transformations [9].

For this reason, the presence of heavy metals in agricultural raw materials and products should be closely monitored. Regulations concerning the maximum allowable levels of those contaminants can differ in various countries. Most of the regulations are laid down by the European Food Safety Authority, and the limits specified by the WHO for the range of the maximum concentrations of Cd, Pb, As, and Hg in herbal raw materials are 0.3, 10.0, 5.0, and 0.2 mg/kg, respectively [10]. Taking into account the health safety and life of the consumers, the present study addressed the subject matter of determination of the content of Cd, Pb, As, and Hg in samples of herbs, spices, tea, and coffee, for the purpose of estimation of the health risk involved in the use of the abovementioned raw materials and products in Polish cuisine.

## 2. Materials and Methods

### 2.1. Sample Collection

The test material used in the study consisted of samples of unprocessed plant materials acquired at random from farms situated in the eastern part of Poland in the period of 2015–2018. Imported spices, tea, and coffee were purchased in supermarkets in Lublin. The samples were taken according to the guidelines contained in Dz.U. 2006 nr 181 poz. 1336 [11]. The minimal weight of a sample was 3 kg. A total of 240 samples were analysed. The samples were classified into four groups:

1. Herbs (163)
  - Green matter: hoary rockrose (*Cistus incanus* L.)—(7), lemon balm (*Melissa officinalis* L.)—(4), common sage (*Salvia officinalis* L.)—(4), common thyme (*Thymus vulgaris* L.) (3), field horsetail (*Equisetum arvense* L.)—(3), heath speedwell (*Veronica officinalis* L.)—(3), common mugwort (*Artemisia vulgaris* L.)—(3), yellow bedstraw (*Galium verum* L.) (3), ground ivy (*Glechoma hederacea* L.)—(3), smallflower hairy willowherb (*Epilobium parviflorum* Schreb.)—(3), southernwood (*Artemisia abrotanum* L.)—(3), common oat (*Avena sativa* L.)—(3);
  - Root: valerian (*Valeriana officinalis* L.)—(17), liquorice (*Glycyrrhiza glabra* L.)—(8), dandelion (*Taraxacum officinale* Web.)—(4), greater burdock (*Arctium lappa* L.)—(3), Japanese knotweed (*Reynoutria japonica* Houtt.)—(3), marshmallow (*Althaea officinalis* L.)—(3), common chicory (*Cichorium intybus* L.)—(3),
  - Leaf: European blueberry (Vaccinium myrtillus L.)—(3), blackcurrant (*Ribes nigrum* L.)—(3), white mulberry (*Morus alba* L.)—(3), globe artichoke (*Cynara scolymus* L.)—(3), red raspberry (*Rubus idaeus* L.)—(3), goutweed (*Aegopodium podagraria* L.)—(3), bear’s garlic (*Allium ursinum* L.)—(3);
  - Flower: blackthorn (*Prunus spinosa* L.)—(3), red clover (*Trifolium pratense* L.)—(3), common heather (*Calluna vulgaris* L.)—(3), hollyhock (*Alcea rosea* L.)—(3), chamomile (*Matricaria chamomilla* L.)—(4), common sunflower (*Helianthus annuus* L.)—(3), small-leaved lime (*Tilia cordata* Mill.)—(3), white nettle (*Lamium album* L.)—(3);
  - Fruit: dog rose (*Rosa canina* L.)—(3), rowan (*Sorbus aucuparia* L.)—(4), midland hawthorn (*Crataegus oxyacantha* L.)—(3), chasteberry (*Vitex agnus-castus* L.)—(3);
  - Seeds: common flax (*Linum usitatissimum* L.)—(24), narrowleaf plantain (*Plantago lanceolata* L.)—(3).
2. Spices (61)
  - Green matter of basil (*Ocimum basilicum* L.)—(3);
  - Rhizome: common onion (*Allium cepa* L.)—(3), common garlic (*Allium sativum* L.)—(3);
  - Root of turmeric (*Curcuma longa* L.)—(3);
  - Flowers of clove tree (*Eugenia caryophyllus* L.)—(3);
  - Fruit: black pepper (*Piper nigrum* L.)—(26), peppers (*Capsicum annuum* L.)—(8), allspice (*Pimenta dioica* L.)—(3);
  - Seeds: cumin (*Cuminum cyminum* L.)—(3), fennel (*Foeniculum vulgare* Mill.)—(3), fenugreek (*Trigonella foenum-graecum* L.)—(3).
3. Leaf of China tea (green) (8).
4. Beans of Arabica coffee (roasted) (8).

Prior to the analyses, the samples tested were homogenised and ground as required (plant samples were milled and sieved through a 0.5 mm mesh), after which the moisture level of each product was measured for conversion to dry matter. The plant material was standardised through pooling together and fragmentation to a homogeneous fraction with the use of a laboratory analytical mill type A11 Basic (IKA), after which, using a moisture analyser type WPS 50 SX (RAD WAG, Poland), the moisture content of every product was determined in the aspect of conversion to dry matter.

### 2.2. Evaluation of the Contents of Cd, Pb, As, and Hg

The content of metals in the analysed samples was determined following microwave mineralisation [12]. Approximately 0.5 g of each plant sample was placed in a Teflon vessel. Then, 10 mL of 65% HNO_3_ (suprapur grade) supplied by (Merck, Darmstadt, Germany) was added to the vessel, and the sealed vessel was put into a microwave mineraliser MARS Express (CEM, Matthews, NC, USA). The microwave mineralisation was performed stepwise at 400 W and 363 K, at 800 W and 393 K, and at 1600 W and 483 K. The cooled digestion solution was then diluted to 50 mL using high-purity deionised water.

### 2.3. ICP–MS

To determine Pb, Cd, and As content in the plant samples, an inductively coupled plasma mass spectrometer ICP-MS 820-MS (Varian, Mulgrave, Australia) with quadrupole mass analyser was used. The instrumental conditions for trace-element determination by ICP–MS were as follows: plasma: argon plasma; plasma flow: 18 L/min; auxiliary flow: 1.8 L/min; stealth gas flow: 0.12 L/min; nebuliser flow: 0.95 L/min; sampling depth: 6 mm; RF power: 1.35 kW; pump rate: 0.1 Hz; stabilisation delay: 35 s; first extraction lens: 5 V; second extraction lens: 190 V; third extraction lens: 225 V; corner lens: 200 V; left mirror lens: 39 V; right mirror lens: 34 V; bottom mirror lens: 36 V; entrance lens: 1.00 V; fringe bias: −2.90 V; entrance plate: −39 V.

All certified single-element standard solutions used to prepare the calibration curve (1000 mg/L) used were of the highest purity grade (99.999%) and were supplied by Ultra Scientific. The blank calibration standards for ICP–MS analysis were prepared by diluting solutions in 1% HNO_3_ and in high-purity deionised water. Each sample, depending on the plant material, was diluted appropriately to the range of the standard curve. The results were expressed in mg/kg of dry matter (DM).

### 2.4. Mercury Analyzer

Mercury was determined independently using a non-flame atomic spectrometry absorption technique (mercury analyser AMA 254, Altec, Czech Republic) according to a previously described method [13]. The original factory calibration was still valid for the calibration of the instrument. The values were controlled regularly by calibration standard mercury solutions—NIST-traceable Hg standard solution (Accu Trace Single Element Standard; AccuStandard Inc., New Haven, CT, USA) [13].

### 2.5. Analytical Quality

During the analysis of Hg, Cd, Pb, and As, the analytical quality was controlled by means of measurement of a blind sample, a double sample, and the certified reference materials: tea leaves (INCT-TL-1) and mixed polish herbs (INCT-MPH-2) (Table 1).

### 2.6. Health Risk Assessment

The health risk assessment (noncarcinogenic hazard) related to the presence of heavy metals in analysed samples was performed using a previously described model [14]. The target hazard quotient (THQ) was used for the calculation of noncarcinogenic hazard of ingestion of heavy metals (Equation (1)) [14,15].

THQ = (EFi × EDi × MSi × C)/(RfD × BWi × AT).(1)

The estimated daily intake EDI (mg analysed element kg/body weight/day) was calculated using the following equation [14,16]:

EDI = (MSi × C)/BWi,(2)

where C is the trace element concentration in canned meat and canned fish (expressed as µg/kg w.w. in EDI and as µg/kg in THQ), MSi is the mass of selected dietary ingested in adults (average daily consumption of investigated products in Poland: spices—0.7 g/day [17], tea—2.7 g/day [18], coffeee—7.8 g/day [19], herbs—0.7 g/day [20]), EFi is the exposure frequency (365 days/year), Edi is the exposure duration (70 years), BWi is the average body weight (70 kg), and AT is the average exposure time for noncarcinogens (365 days/year × ED).

When THQ >1, there is a probability of potentially harmful effects occurring, while, when THQ ≤1, there is no probability of unfavourable effects [15].

RfD is the heavy metal oral intake reference dose (mg/kg/day). The RfDs for lead, mercury, cadmium, and arsenic are 0.0036, 0.0003, 0.001, and 0.0003 mg/kg/day, respectively [21,22,23].

In order to estimate total target hazard quotient (TTHQ) for multiple heavy metals, the sum of THQi for individual heavy metals was estimated using Equation (3) [15,21].

(3) $$\mathrm{TTHQ}=\overset{n}{\underset{i=1}{\sum }}\mathrm{THQi}.$$

As an estimate, carcinogenic risk (CR) was calculated using Equation (4) [15,22].

CR = EDI × SF,(4)

where CF is the cancer slope factor (mg/kg/day), i.e., the probability of the substance to increase cancer risk via the oral exposure route. CR is estimated as the incremental probability of an individual developing cancer over a lifetime. For example, a CR of 10^−4^ indicates a probability of 1 in 10,000 individuals developing cancer. Risks in the range of 1.0 × 10^−6^ to 1.0 × 10^−4^ are acceptable [15,22].

Arsenic was treated as a potential carcinogenic contaminant, whereas lead and mercury were regarded as noncarcinogenic elements, according to the order of the classification group defined by the International Agency for Research on Cancer [24]. The CF for arsenic is 1.5 mg/kg/day [25]. The CFs for Cd, Hg, and Pb were not available. In this study, the value of estimated CR was compared with the maximum acceptable risk suggested by the USEPA, which is ≤1.0 × 10^−4^ [22].

## 3. Results and Discussion

The results of the analysis of heavy metals in selected samples of herbs, spices, tea, and coffee are presented in Table 2. To determine the degree of contamination of the analysed samples with heavy metals, the presented results were referenced to the guidelines of the World Health Organisation (WHO) and the Commission Regulation (EU) No. 420/2011, setting maximum allowable levels of certain contaminants in fresh herbs, in conversion to dry matter, with the assumption of water content at the level of 90%.

### 3.1. Cadmium

In the analysed samples of herbs, the content of cadmium was diversified in relation to the species and varied within the range from <LOQ (0.020 mg/kg) to 2.170 mg/kg. Among the 163 analysed samples of herbs, in 24 samples (15%), the assayed cadmium levels exceeded the limit values set by the World Health Organisation (WHO) at the level of 0.3 mg/kg [10]. Excessive values were noted for nine herbal species—green matter of hoary rockrose, heath speedwell, yellow bedstraw, southernwood, root of marshmallow, leaves of globe artichoke and red raspberry, flower of hollyhock, and fruit of midland hawthorn. In accordance with the guidelines of the Commission Regulation (EU) No. 420/2011 setting maximum allowable levels of certain contaminants in fresh herbs, in conversion to dry matter, in one sample of the cistus herb, it was found that the allowable cadmium content was exceeded—2.17 mg/kg. In 41 samples (25%) of 12 species of herbs (green matter of field horsetail, root of liquorice and dandelion, leaves of white mulberry and goutweed, flower of red clover, common heather, common sunflower and white nettle, fruit of dog rose and chasteberry, and seeds of narrowleaf plantain), no presence of cadmium was found in any of the analysed samples. The lowest levels of cadmium, within the range from 0.020 to 0.333 mg/kg, were noted in samples of green matter of lemon balm, common sage, common thyme, ground ivy, common oat, and smallflower hairy willowherb, root of valerian, greater burdock, Japanese knotweed, and common chicory, leaves of European blueberry, and bear’s garlic, flowers of blackthorn, camomile, and small-leaved lime, fruit of rowan, and seeds of common flax. The highest concentrations of cadmium were assayed in samples of green matter of hoary rockrose and common mugwort and leaves of globe artichoke, at 2.170 mg/kg, 1.670 mg/kg, and 0.654 mg/kg, respectively.

The scope of the presented study also included the estimation of cadmium concentration in 61 samples of spices used in Poland. The level of contamination of the analysed samples with cadmium was low and varied from <LOQ (0.020 mg/kg) to 0.082 mg/kg. None of the analysed samples contained cadmium levels exceeding the permitted limits for that metal. In all analysed samples of five species of spices (green matter of basil, turmeric, fruit of allspice, seeds of fennel, and flowers of clove tree), no contamination with cadmium was noted. The highest average concentrations of cadmium were noted in samples of black pepper and seeds of cumin, at 0.080 and 0.082 mg/kg, respectively. 

Another group of samples analysed within the scope of this study for the level of contamination with cadmium included eight samples of leaves of China tea and eight samples of Arabica coffee beans. The analyses revealed low levels of that element in the samples, within the range from <LOQ (0.020 mg/kg) to 0.098 mg/kg.

The results obtained in this study are comparable to most of the results published in the literature. The authors of the cited publications obtained similar levels of cadmium in samples of herbs and spices analysed in Poland. Mirosławski and Pakuszto [26] analysed herbs (leaf of peppermint and flower of camomile) available on the Polish market and found average levels of cadmium varying from 0.180 mg/kg to 0.730 mg/kg. Similar results concerning the level of heavy metals in selected Polish herbs and spices (green matter of basil, root of curcuma, flower buds of clove tree, fruit of black pepper and peppers, onion, bear’s garlic, and allspice) were obtained by Krejpcio et al. [27], who reported that the average content of cadmium varied within the range from 0.010 to 0.140 mg/kg. Gajewska et al. [28] analysed herbal plants and spices available on the retail market in Poland and demonstrated average cadmium levels of 0.011–0.879 mg/kg. Low levels of cadmium in samples of spices available in Poland were confirmed in a study by Staniek and Krejpcio [29], who reported that the accumulation of cadmium in samples of peppers and black pepper varied from 0.015 to 0.017 mg/kg. Fischer et al. [30] analysed spice plants grown in Poland and noted cadmium concentrations within the range from 0.02 to 1.94 mg/kg.

The presented results correspond with analyses of samples of herbs and spices conducted in other European countries. A monitoring study on the content of heavy metals conducted in Austria [31] demonstrated that the level of cadmium in herbs and spices was at levels from 0.02 mg/kg in samples of green matter of lemon balm and common yarrow to 0.98 mg/kg in green matter of St John’s wort. Reinholds et al. [32] analysed herbs and spices and noted diversified levels of cadmium dependent on the plant species. They demonstrated the highest accumulation of cadmium in samples of green matter of common thyme and basil, at 0.400 mg/kg and 0.120 mg/kg, respectively, while the lowest levels of that element were noted in samples of black pepper and wild marjoram—0.010 mg/kg and 0.020 mg/kg, respectively.

Analyses of herbal raw materials from other regions of the world show similar results to those obtained in the presented study. Literature data from Turkey concerning the study of the levels of heavy metals reported cadmium contamination of samples of herbs and spices (green matter of common sage, basil, common thyme, lemon balm, flower of small-leaved lime and camomile, fruit of midland hawthorn and dog rose, and seeds of fennel) in the range from 0.010 mg/kg to 2.700 mg/kg [33,34,35]. Studies on the content of heavy metals conducted in Iran and Iraq noted cadmium levels in analysed samples of herbal plants and spices in the range from 0.012 mg/kg in samples of curcuma to 1.64 mg/kg in samples of dill [36,37,38]. In the United Arab Emirates, Dghaim et al. [39] analysed seven species of herbal plants (parsley, basil, common sage, oregano, mint, common thyme, and camomile) and demonstrated average levels of cadmium in the range from less than 0.1 mg/kg in green matter of mint to 1.11 mg/kg in green matter of basil. Dghaim et al. [39] also demonstrated that 55% of samples of camomile inflorescences contained cadmium (0.82 mg/kg) in excess of the highest permissible level (0.3 mg/kg), which was never found in the results presented in this study. In selected studies on the accumulation of cadmium in samples of herbal plants and spices, decidedly higher levels of cadmium content were noted, in the range of 0.23 mg/kg to 23.1 mg/kg [40,41,42], considerably exceeding the WHO limits for that metal, which was not shown in the presented results of our study.

The results obtained in this study, indicating low levels of cadmium in samples of tea, are in support of earlier studies in this area. Wojciechowska and Mazurek [43] analysed Indian teas available on the Polish market and found average cadmium levels of 0.034 mg/kg in samples of leaf tea and 0.026 mg/kg in samples of tea bags. Another study, focusing on the analysis of herbal teas produced in Poland, indicated contamination with that metal in the range of 0.024–0.153 mg/kg [44]. Winiarska-Mieczan et al. [45] also analysed Polish herbal teas and noted an average cadmium level of 0.2 mg/kg. Studies on the accumulation of cadmium in various kinds of tea, conducted by Sarojama [46] and by Zhong et al. [47], revealed contamination of the analysed samples at levels from 0.021 mg/kg to 0.064 mg/kg and from 0.010 mg/kg to 0.390 mg/kg, respectively. The lowest level of cadmium was noted in black tea, while the highest level was assayed in Pu’erh tea. The content of cadmium in samples of green tea varied from 0.040 to 0.110 mg/kg [47]. Basgel and Erdemoglu [48] analysed herbal teas and demonstrated cadmium levels in the range from 0.004 mg/kg for dill tea to 0.440 mg/kg for camomile tea. Certain literature reports highlighted decidedly higher levels of cadmium contamination of tea samples, i.e., from 16.7 mg/kg to 69.85 mg/kg [49], which are not in line with the presented research results.

The low levels of cadmium contamination of samples of coffee noted in the presented study correspond with the results of a Polish study by Adler et al. [50], who analysed samples of coffee and demonstrated that the content of cadmium varied from 0.014 mg/kg to 0.015 mg/kg in green coffee beans and from 0.011 mg/kg to 0.022 mg/kg in roasted beans of coffee. Długaszek et al. [51] analysed the content of cadmium in Colombian coffee purchased in Poland and noted its accumulation at a level similar to that observed in the presented study. Similar results of cadmium content, within the range from 0.025 mg/kg to 0.100 mg/kg, were obtained in a study on Brazilian coffee [52]. In a study by Grembecka et al. [53], cadmium content in roasted coffee beans from Africa, America, Asia, and Oceania oscillated below the level of detection.

### 3.2. Lead

In the analysed samplers of herbs, the level of lead was diversified in relation to the species and varied within the range from <LOQ (0.010 mg/kg) to 5.680 mg/kg. The content of lead was not in excess of the limit value set by the World Health Organisation (WHO) at the level of 10 mg/kg in any of the analysed samples of herbs. Among the 163 analysed samples, no presence of lead was detected (green matter of common thyme and field horsetail, leaf of globe artichoke, and fruit of chasteberry) in only 12 samples (7%). The lowest average levels of lead, ranging from 0.022 mg/kg to 0.201 mg/kg, were noted in samples of seven herbal species—green matter of common oat, root of common chicory, leaf of white mulberry, flower of common sunflower, fruit of rowan, and seeds of common flax and narrowleaf plantain. The highest levels of contamination with lead were noted in samples of root of valerian and green matter of lemon balm and common sage, at 5.680 mg/kg, 3.47 mg/kg, and 2.550 mg/kg, respectively.

Analysis of the content of lead in 61 samples of spices available on the Polish market demonstrated diversified levels of the analysed metal, in relation to the plant species (<LOQ = 0.010–1.92 mg/kg). In 97% of the analysed samples of spices, contamination with lead was noted; however, the assayed levels of lead were not in excess of the permissible limits for this metal in any of the samples. The lowest lead content, ranging from 0.069 to 0.202 mg/kg, was noted in samples of four herbal species—bear’s garlic, black pepper, peppers, and fenugreek. The highest concentration of lead, at the level of 1.92 mg/kg, was assayed in samples of green matter of basil.

Analyses of lead contamination of samples of leaves of Chinese tea and beans of Arabica coffee presented in this study demonstrated low levels of the metal in the analysed samples, ranging from less than 0.010 mg/kg to 0.791 mg/kg.

The results obtained in this study, concerning lead contamination of samples of herbs and spices, correspond with earlier studies on this subject matter, conducted in Poland. Mirosławski and Pakuszto [26] analysed herbs (leaf of peppermint and flower of camomile) available on the Polish market and noted average levels of lead in the range of 0.440 mg/kg–1.110 mg/kg. Similar results concerning the levels of Pb in selected Polish herbs and spices (green matter of basil, root of curcuma, flower buds of clove tree, fruits of black pepper and peppers, onion, bear’s garlic, and allspice) were obtained by Krejpcio et al. [27], who reported that the average content of lead varied in the range from 0.250 mg/kg to 1.490 mg/kg. Gajewska et al. [28], in a study on herbal plants and spices available on the retail market in Poland, demonstrated average levels of lead in the range of 0.020 mg/kg–2.25 mg/kg. The low levels of lead in samples of spices available in Poland find support in a study by Staniek and Krejpcio [29], who reported that the accumulation of lead in samples of peppers and black pepper varied in the range from 0.044 to 0.377 mg/kg.

The presented results correspond also with analyses of herbal and spice samples conducted in other European countries, e.g., in Serbia [54], in which the concentration of lead in samples of black pepper varied within the range from 0.280 mg/kg to 0.42 mg/kg. A monitoring study concerning the level of heavy metals in products in Austria [31] demonstrated that the level of lead in herbs and spices varied within the range from 0.2 mg/kg in samples of the fruit of caraway to 1.9 mg/kg in leaves of lovage. Reinholds et al. [32] analysed herbs and spices and noted diversified levels of lead, depending on the plant species. They assayed the highest accumulation of lead in samples of green matter of common thyme and basil, at 0.790 mg/kg and 0.480 mg/kg, respectively, and the lowest in samples of nutmeg, black pepper, and oregano, from 0.130 mg/kg to 0.280 mg/kg, respectively. Furthermore, in our study the highest concentration of lead was assayed in samples of green matter of basil, while the lowest levels of this metal were noted in samples of black pepper. Literature data from Turkey, concerning the levels of heavy metals, report a similar level of lead contamination of samples of herbs and spices (green matter of common sage, basil, common thyme, lemon balm, flower of small-leaved lime, and camomile, fruit of midland hawthorn and dog rose, and seeds of fennel), ranging from 0.1 mg/kg to 4.43 mg/kg [33,34,35].

Numerous literature reports, however, indicated significantly higher levels of lead contamination of analysed samples of herbal raw materials and spices, exceeding the maximum allowable levels set for this metal by the WHO—10 mg/kg [10], which was not demonstrated in the presented study. Notably higher concentrations of lead were presented in a study by Huremović et al. [55], covering six species of spices (black pepper, basil, oregano, peppers, parsley, and rosemary), in which the concentration of the metal varied from 0.740 mg/kg to 20.35 mg/kg. In the study by Huremović et al. [55], nearly 67% of the analysed samples had lead levels exceeding the maximum allowable value for that metal (10 mg/kg), which was not noted in the results of the presented study. Analysis of the content of heavy metals in herbs and spices, conducted in Iran and Iraq, revealed lead contamination in the analysed samples at levels from 0.15 mg/kg in samples of chasteberry to 14.6 mg/kg in samples of fenugreek [37,38]. Dghaim et al. [39] analysed seven herbal plant species consumed in the United Arab Emirates (parsley, basil, common sage, oregano, mint, common thyme, and camomile) and noted average lead levels from less than 1.0 mg/kg in green matter of mint to 23.52 mg/kg in green matter of common thyme. Dghaim et al. [39] also demonstrated that 44% of camomile samples and 100% of samples of common sage contained lead at concentrations exceeding the maximum allowable level, which differs considerably from the results obtained in this study. High levels of lead in samples of herbs and spices, considerably in excess of the limits set by the WHO for this metal, were also demonstrated in studies conducted in Jordan (0.2–41.18 mg/kg), Punjab (3.21–28.7 mg/kg), and India (13.0–54.47 mg/kg) [41,42,56]. Studies on herbal raw materials from various regions of the world have indicated that the content of heavy metals in herbal and spice plants depends on numerous factors, such as the region of their origin, the level of environmental contamination, the plant species, and the technological process.

The results of this study, concerning low levels of lead in samples of leaves of Chinese tea, find support in earlier studies published in the area of this subject matter. Wojciechowska and Mazurek [43] analysed Indian teas available on the Polish market and noted average lead levels of 0.36 mg/kg in samples of leaf tea and 0.29 mg/kg in samples of tea bags. In Poland, Brzezicha-Cirocka et al. [3], as well as Gutt et al. [57], also demonstrated low levels of contamination with the analysed metal in samples of green, black, herbal, and fruit teas. Another study aimed at the analysis of the content of lead in herbal teas produced in Poland demonstrated a similar level of contamination of analysed samples with that element, ranging from 0.44 to 0.77 mg/kg [44].

Literature reports from other parts of the world correspond with the results presented earlier. In a study conducted by Zhong et al. [47], the level of lead in various kinds of Chinese teas varied from 0.48 mg/kg (in samples of jasmine tea) to 10.57 mg/kg (in samples of green tea). Othman et al. [58] noted average concentrations of lead in green tea products imported from China ranging from 0.231 mg/kg to 4.460 mg/kg. Similarly, Basgel and Erdemoglu [48], in a study on herbal teas, demonstrated lead levels within the range from 0.26 mg/kg in the case of inflorescences of small-leaved lime to 4.80 mg/kg in samples of nettle leaves. Those values did not exceed the permissible limits for that metal, and they correspond with the results obtained in the presented study.

The low level of lead contamination of coffee bean samples in the results of the presented study correspond with those obtained in another Polish study, conducted by Adler et al. [50], who demonstrated low levels of concentration of that metal, from 0.008 mg/kg to 0.144 mg/kg in green coffee beans and from 0.013–0.022 mg/kg in roasted coffee beans. Długaszek et al. [51] estimated the content of lead in Colombian coffee purchased in Poland and noted an accumulation of that metal at levels from 0.020 mg/kg to 0.060 mg/kg. Similar results concerning the content of lead, ranging from 0.075 mg/kg to 1.575 mg/kg, were noted in a study on Brazilian coffee [52].

### 3.3. Arsenic

Among the 163 analysed samples of herbs, the presence of arsenic was found only in 12 samples (7%)—in nine samples of the root of valerian (0.111–0.535 mg/kg) and in three samples of leaves of blackcurrant (0.166–0.202 mg/kg). In the remaining 93% of the analysed samples of herbs, the concentration of arsenic was below the threshold of detection (0.1 mg/kg). In the group of 61 analysed samples of spices, arsenic was detected only in five samples of black pepper (0.102–0.863 mg/kg) and in four samples of peppers (0.113–0.178 mg/kg). In all of the analysed samples of coffee beans and tea leaves, the assayed content of the metal in question was below the threshold of detection.

Literature data covering studies in the area of contamination with arsenic support the results obtained in this study. In a study on herbal raw materials, conducted by Alhusbaba et al. [56], no arsenic contamination was demonstrated in any of the 40 analysed samples. Reinholds et al. [32] analysed herbs and spices in the aspect of arsenic contamination of samples and found low average levels of the metal, ranging from 0.03 mg/kg and 0.06 mg/kg in samples of nutmeg and black pepper to 0.37 mg/kg in samples of oregano. A similar level of contamination with arsenic, with an average of 0.053 mg/kg in samples of black pepper, was obtained in the presented study. An Indian study monitoring the content of arsenic in samples of herbal raw materials revealed concentrations of the analysed metal that ranged from below the threshold of detection in samples of long pepper (0.050 mg/kg) to 0.52 mg/kg in samples of ginger [59].

The results obtained in the presented study, concerning the level of contamination with arsenic, find support in a Malaysian study on herbs and spices commonly consumed in that country [40], in which the levels of the analysed metal varied in the range from 0.24 mg/kg in samples of bear’s garlic to 2.54 mg/kg in Philippine flower wax. Similar results of arsenic contamination were presented in a study involving the analysis of various samples of black pepper available on the Serbian market, in which arsenic was assayed in the range from 0.22 mg/kg in samples of green pepper to 0.51 mg/kg in samples of black pepper [54]. Higher levels of contamination of herbal raw materials were reported in studies conducted in Turkey [34] and Iran [36], in which the concentration of arsenic varied from 0.20 mg/kg in samples of dog rose to 19.4 mg/kg in samples of aniseed.

In contrast to our study, in which no detectable residues of the analysed metal were found in samples of tea leaves and coffee beans, a study conducted by Mania et al. [59] on various kinds of tea available on the Polish market indicated that the content of arsenic varied in the range from 0.085 mg/kg in samples of black tea to 0.134 mg/kg in samples of green tea. The level of arsenic contamination in the presented research results (<LOQ = 0.1 mg/kg) was similar to that obtained in Poland [60] and in other EU countries (0.066 mg/kg) [61], but significantly lower than the results obtained in studies conducted in Asia, in which the content of arsenic in various kinds of tea varied in the range from below the threshold of detection to 4.81 mg/kg [62,63,64].

### 3.4. Mercury

The concentration of mercury in the analysed samples of herbs varied in the range from below the threshold of detection (0.005 mg/kg) to 0.030 mg/kg. The presence of mercury was demonstrated in 45 of the analysed samples of herbs (28%), while, in 72% of the analysed samples, the content of mercury was below the threshold of detection equal to 0.005 mg/kg. The lowest content of mercury, at the level of 0.006 mg/kg, was noted in samples of six species of herbs—common mugwort, smallflower hairy willowherb, valerian, marshmallow, common chicory, and blackthorn. The highest levels of contamination with mercury were demonstrated in samples of green matter of field horsetail and in leaves of European blueberry, at 0.020 mg/kg and 0.030 mg/kg, respectively. In all 61 analysed samples of spices, the level of contamination with mercury was below the threshold of detection. In the group of analysed samples of tea leaves and coffee beans, trace levels of mercury were assayed, in the range from below 0.005 mg/kg to 0.007 mg/kg.

In the area of contamination of samples of herbal raw materials with mercury, the results of the presented study correspond with the results obtained by Fischer et al. [65], who analysed the level of contamination of selected herbs grown in Poland and demonstrated levels of the analysed metal in the range from 0.005 mg/kg in samples of green matter of St. John’s wort to 0.025 mg/kg in samples of nettle leaves. Mercury contents at levels from below 0.005 mg/kg to 0.020 mg/kg were also noted by Kowalski and Kucharski [66] in a study on herbal raw materials available on the Polish market. An Asian study on over a dozen herbal raw materials popular in Korea also revealed an absence of contamination with mercury in 100% of the analysed samples of herbs [67]. Similar results in this respect were presented in a study on herbal samples, conducted in India, in which no contamination with mercury was assayed in 90% of analysed samples, with the exception of one sample of liquorice, in which the level of mercury was 0.060 mg/kg [59].

The low levels of mercury in the analysed samples of spices find support in a Serbian study on various kinds of pepper [54], in which the level of contamination of samples with the analysed metal was 0.103 mg/kg in samples of green pepper, while, in the remaining samples of black and white pepper, contamination with mercury was below the threshold of detection (0.003 mg/kg). In an Indian study, no contamination with mercury was noted in all analysed samples of black pepper, curcuma, and cumin [41], which is in line with the results obtained in the presented study.

Selected literature reports concerning the level of contamination of samples of herbal raw materials with heavy metals presented higher levels of mercury in analysed samples. In a study on spices and herbs commonly consumed in Malaysia [40], the authors noted mercury contamination levels in the range from 0.06 mg/kg in samples of leaves of kaffir lime to 0.52 mg/kg in a sample of ginger. The differences in the levels of contamination of samples of herbs and spices with heavy metals reflect the great diversity in the level of contamination of local environments with the analysed metals (in this case, mercury).

The trace amounts of mercury in samples of tea leaves and coffee beans reported in the presented study are in support of an earlier study conducted in this field by Kowalski et al. [13]. Among 81 analysed samples of fruit teas, teas regulating digestion, green teas, black teas, and Pu’erh type teas, the presence of mercury was assayed at a level above 0.02 mg/kg in only one sample. The cited authors demonstrated the highest concentration of mercury in one sample of a single-component fruit tea (0.031 mg/kg). In the case of green teas, the highest concentration of mercury was 0.007 mg/kg, and, in the case of black teas, the maximum assayed level of mercury was 0.009 mg/kg. Wojciechowska-Mazurek et al. [43] reported that the average contamination of leaf teas, tea bags, granulated teas, and fruit teas was 0.008 mg/kg, 0.007 mg/kg, 0.009 mg/kg, and 0.003 mg/kg, respectively, which is generally in line with the presented study. Similar results concerning the contamination of samples of leaf tea with mercury, at the level of 0.005 mg/kg, were reported by Forsberg and McQuatters [68]. A study conducted by the Canadian Food Inspection Agency in the area of the content of mercury in samples of tea revealed that 53% of the analysed samples had no detectable residues of mercury, while, in the remaining 47% of the analysed samples, the highest assayed concentration of mercury was 0.023 mg/kg [69].

### 3.5. Health Risk Assessment (Noncarcinogenic Risk)

The THQ rank order of heavy metals in spices was As > Cd > Pb > Hg (Table 3). In the case of herbs, the THQ was characterised by higher values compared to spices for Pb, Hg, and Cd, while, in the case of As, the value was lower, i.e., Cd > As > Pb > Hg. The values of THQ for leaves of Chinese tea could be ranked as follows: Cd > Pb > Hg > As. The series of values of THQ calculated for roasted coffee beans were as follows: Cd > Pb > Hg > As.

As the concentration of Cd was generally fairly high, relative to the other toxic elements (Table 2), and its RfD value was also relatively low (0.001 mg/kg/day) [22], the THQ value for Cd was higher than that for the other monitored levels of elements. The values of TTHQmax (in relation to the consumption of the analysed products) were up to 4.23 × 10^−2^ for spices, up to 2.51 × 10^−1^ for herbs, up to 4.03 × 10^−2^ for Chinese leaf tea, and up to 1.25 × 10^−1^ for roasted coffee beans (Table 2). Since the value of THQ ≤ 1, there was no probability of occurrence of unfavourable effects [15]. According to the available literature data, THQ values for heavy metals in herbs, spices, tea, and coffee were considerably lower than 1. As an example, in a study conducted in Iran, the calculated values of THQ related to the consumption of herbs were as follows: in the case of Cd—up to 1.50 × 10^−2^, in the case of Pb—in the range up to 1.60 × 10^−2^ [36]; in another study, the values were as follows: in the case of Cd—up to 1.49 × 10^−1^, As—up to 8.40 × 10^−2^, Pb—up to 3.00 × 10^−2^, Hg—7.00 × 10^−3^ [36]. Similarly low values of THQ were obtained by authors of other studies. In Egypt, in a study on spices, the values for As were up to 7.50 × 10^−2^ [70]; in a study conducted in Ethiopia, the values obtained for Pb were up to 30.75 × 10^−2^ [71]; in a study on herbal products, conducted in Ghana, the following values of THQ were obtained: in the case of As—up to 8.28 × 10^−2^, for Cd—up to 0.0015, for Pb—up to 0.0034, and for Hg—up to 0.0001 [72]. In another study conducted in Ghana, the values of THQ in the case of absorption of heavy metals related with the consumption of tea were as follows: for As—up to 0.89, for Pb—up to 0.0109, and for Cd—up to 2.00 × 10^−1^ [73]. Total target hazard quotient (TTHQ) values for all samples of spices analysed in Nigeria fell within the range of 6.00 × 10^−2^–5.00 × 10^−1^ [74]. On the other hand, THQ values determined for metals analysed in a study conducted in China were as follows: for Pb—up to 1.24 × 10^−2^, Cd—up to 1.20 × 10^−2^, As—up to 4.53 × 10^−2^, and Hg—up to 6.00 × 10^−2^ [75]. In a Polish study, THQ values related to the consumption of coffee were up to 2.33 × 10^−1^ and up to 6.70 × 10^−2^ for Pb and Cd, respectively [76].

CR values of As were from 0 (tea and coffee mean value of As) to 1.95 × 10^−6^ (spices mean value of As) and from 0 (tea and coffee max. value of As) to 1.29 × 10^−5^ (spices max. value of As) (Table 4). All values determined for the carcinogenic risk were at the level lower than the maximum acceptable level of 1.0 × 10^−4^, which indicates no significant influence on this risk according to the United States Environmental Protection Agency (USEPA).

The presented results and the current toxicological standards concerning the health risk related with the consumption of the analysed group of raw materials and products of plant origin in Poland do not indicate any concern resulting from the introduction of heavy metals, together with the diet, into the human organism. However, does the present state of knowledge allow stating without a doubt that the adopted toxicological standards are correct, especially as data on the effects of poisoning may appear after a long period of dormancy? In addition, the study indicates that, in particular, plant products originating in their majority from local production do not indicate any potential contamination of natural or agricultural areas with toxic elements.

Results published by us earlier, as well as those presented in this study, indicate that it is necessary to conduct research that would allow the estimation of the content of toxic elements in food products which have a significant contribution to our diet and could cause potential health problems [77,78].

The contents of heavy metals in plant varied according to the character of soil, the pollution resource distribution, and the species of plant. Thus, the metal content level should be discussed taking into account the environmental condition. A dangerous phenomenon is the accumulation of heavy metals in the soil, which accumulate in the surface layer. The high concentration of heavy metals contributes to the degradation of the chemical properties of soils and to the contamination of ground and surface waters. When heavy metals are present in the soil in a dissolved form, they are absorbed by plants and accumulated in plant tissues. The most common source of soil contamination with heavy metals is represented by mineral fertilisers (mainly phosphorus fertilisers, followed by calcium, potassium, and nitrogen fertilisers) [79]. Monitoring studies from 1995–2010 showed no excessive contamination of soils in the Lublin region with heavy metals (including lead and cadmium). The increased levels of some metals observed locally did not significantly affect soil properties. No metal accumulation was observed in the soil surface layer for any of the heavy metals. [80].

Other results also confirmed the low level of heavy metals in the soil from arable land located along the busy section of the national road DK63 Siedlce–Łuków, particularly exposed to the effects of factors increasing the risk of heavy metals. In the studied soils located near the DK63 national road, the authors did not find any contamination with heavy metals. Regardless of the point of soil sampling, the amount of these elements was within the range of geochemical background values and did not exceed the permissible concentrations for agricultural use provided for in the Regulation of the Minister of the Environment, and it was within the range of natural content, developed by the Institute of Soil Science and Plant Cultivation (IUNG-PIB) in Puławy [80]. In another study, the total content of, among others, lead in the light soils of the Roztocze National Park buffer zone was determined. It is well known that, in light, usually acidic soils with a poor sorption complex, the negative effect of phytotoxic compounds, such as heavy metals, is most often revealed. The authors of these studies showed that the permissible values of heavy-metal concentrations were not exceeded in soils. Soils with such a heavy-metal content can be used for agricultural production without restrictions [81]. Other studies also confirmed that the content of heavy metals in the soils of the eastern region of Poland was relatively low and did not exceed the relevant standards [82,83].

## 4. Conclusions

Summing up the results of the presented study, we can conclude that lead was the heavy metal assayed in the analysed samples with the highest concentrations. Its levels varied in the range from 0.010 to 5.680 mg/kg. The element which was assayed in the analysed samples with the smallest amounts was mercury. Its content varied in the range from 0.005 to 0.030 mg/kg. The level of contamination of the analysed samples with the toxic metals followed the decreasing sequence of Pb > Cd > As > Hg. A decidedly higher accumulation of the analysed heavy metals was noted in the analysed samples of herbs and spices (0.005–5.680 mg/kg), compared to the samples of tea and coffee (0.005–0.791 mg/kg). In reference to the guidelines of the World Health Organisation (WHO) concerning the limits of contamination of samples of herbal raw materials with heavy metals, lead concentrations in excess of the limit values were noted in only 24 samples of herbs (18%). In all of the analysed samples of spices, tea, and coffee, no instances of exceeded maximum permitted levels were found for any of the analysed heavy metals. In the case of Cd, the value of THQ was higher than that for the other monitored elements. Since the value of THQ ≤ 1, there was no probability of occurrence of unfavourable health-related effects caused by the consumption of the analysed group of raw materials and products of plant origin.

## Figures and Tables

**Table 1:** Validation parameters of the analytical procedure for the determination of Cd, Pb, As, and Hg.

Validation Parameters	Metals			
	Hg	Cd	Pb	As
LOD * (mg/kg)	0.002	0.01	0.005	0.05
LOQ ** (mg/kg) *	0.005	0.02	0.01	0.1
Linearity	0.9999	0.9999	0.9999	0.9999
Working range (mg/kg)	0.005–5	0.02–2	0.01–5	0.1–10
Reproducibility (%)	3.80	2.42	6.07	3.18
Recovery (%)	90	99	99	101
Expanded uncertainty (%)	24	6	12	7

Hg (mercury), Cd (cadmium), Pb (lead), As (arsenic) Validation parameters were determined on the basis of 20 repetitions. * LOD—limit of detection. ** LOQ—limit of quantitation.

**Table 2:** The heavy-metal contents in selected samples of herbs, spices, tea, and coffee.

Parts Used	Botanical Name	Samples Analysed	Cd (mg/kg)	Pb (mg/kg)	As (mg/kg)	Hg (mg/kg)
Herbs						
Herbs	Hoary rockrose *Cistus incanus* L.	7	<LOQ = 0.02–2.17 0.935	<LOQ = 0.01–2.01 0.979	<LOQ = 0.1	<LOQ = 0.005–0.016 0.009
	Lemon balm *Melissa officinalis* L.	4	0.026–0.040 0.034	0.531–3.47 1.91	<LOQ = 0.1	<LOQ = 0.005
	Common sage *Salvia officinalis* L.	4	<LOQ = 0.02–0.035 0.009	1.96–2.55 2.21	<LOQ = 0.1	<LOQ = 0.005
	Common thyme *Thymus vulgaris* L.	3	0.102–0.147 0.125	<LOQ = 0.01	<LOQ = 0.1	<LOQ = 0.005
	Field horsetail *Equisetum arvense* L.	3	<LOQ = 0.02	<LOQ = 0.01	<LOQ = 0.1	0.007–0.020 0.015
	Heath speedwell *Veronica officinalis* L.	3	0.399–0.485 0.453	0.99–1.15 1.07	<LOQ = 0.1	<LOQ = 0.005
	Common mugwort *Artemisia vulgaris* L.	3	1.39–1.67 1.53	0.482–0.601 0.554	<LOQ = 0.1	0.006–0.008 0.007
	Yellow bedstraw *Galium verum* L.	3	0.380–0.481 0.434	0.189–0.246 0.209	<LOQ = 0.1	<LOQ = 0.005
	Ground ivy *Glechoma hederacea* L.	3	0.157–0.201 0.183	0.295–0.405 0.359	<LOQ = 0.1	0.009–0.017 0.012
	Smallflower hairy willowherb *Epilobium parviflorum* Schreb.	3	<LOQ = 0.02–0.119 0.040	0.328–0.588 0.460	<LOQ = 0.1	0.006–0.007 0.006
	Southernwood *Artemisia abrotanum* L.	3	0.295–0.427 0.368	1.20–1.85 1.57	<LOQ = 0.1	<LOQ = 0.005
	Common oat *Avena sativa* L.	3	<LOQ = 0.02–0.053 0.018	0.022–0.343 0.197	<LOQ = 0.1	<LOQ = 0.005
Rhizome	Valerian *Valeriana officinalis* L.	17	<LOQ = 0.02–0.333 0.117	0.120–5.68 1.730	<LOQ = 0.1–0.535 0.127	<LOQ = 0.005–0.013 0.003
	Liquorice *Glycyrrhiza glabra* L.	8	<LOQ = 0.02	0.174–0.334 0.270	<LOQ = 0.1	<LOQ = 0.005
	Dandelion *Taraxacum officinale* Web.	3	<LOQ = 0.02	0.299–0.407 0.337	<LOQ = 0.1	<LOQ = 0.005
	Greater burdock *Arctium lappa* L.	3	<LOQ = 0.02–0.316 0.189	0.36–0.46 0.41	<LOQ = 0.1	<LOQ = 0.005
	Japanese knotweed *Reynoutria japonica* Houtt.	3	0.052–0.128 0.077	0.28–0.35 0.30	<LOQ = 0.1	<LOQ = 0.005
	Marshmallow *Althaea officinalis* L.	3	0.220–0.461 0.340	0.258–0.389 0.331	<LOQ = 0.1	0.006–0.007 0.006
	Common chicory *Cichorium intybus* L.	3	0.075–0.124 0.091	0.073–0.201 0.116	<LOQ = 0.1	0.006–0.007 0.006
Leaves	European blueberry *Vaccinium myrtillus* L.	3	0.202–0.322 0.256	1.23–1.99 0.60	<LOQ = 0.1	0.010–0.030 0.02
	Blackcurrant *Ribes nigrum* L.	3	0.223–0.382 0.287	0.198–0.281 0.230	0.166–0.202 0.184	<LOQ = 0.005
	White mulberry *Morus alba* L.	3	<LOQ = 0.02	<LOQ = 0.01–0.20 0.067	<LOQ = 0.1	<LOQ = 0.005–0.015 0.009
	Globe artichoke *Cynara scolymus* L.	3	0.554–0.654 0.603	<LOQ = 0.01	<LOQ = 0.1	<LOQ = 0.005
	Red raspberry *Rubus idaeus* L.	3	0.211–0.613 0.345	0.242–0.507 0.330	<LOQ = 0.1	<LOQ = 0.005–0.010 0.007
	Goutweed *Aegopodium podagraria* L.	3	<LOQ = 0.02	0.482–0.599 0.528	<LOQ = 0.1	0.007–0.009 0.008
	Bear’s garlic *Allium ursinum* L.	3	0.020–0.040 0.030	0.382–0.450 0.416	<LOQ = 0.1	0.007–0.009 0.008
Flowers	Blackthorn *Prunus spinosa* L.	3	0.049–0.061 0.054	1.282–1.421 1.341	<LOQ = 0.1	0.006–0.007 0.006
	Red clover *Trifolium pratense* L.	3	<LOQ = 0.02	0.322–0.462 0.384	<LOQ = 0.1	<LOQ = 0.005
	Common heather *Calluna vulgaris* L.	3	<LOQ = 0.02	0.402–0.502 0.462	<LOQ = 0.1	<LOQ = 0.005
	Hollyhock *Alcea rosea* L.	3	0.392–0.552 0.465	0.254–0.345 0.299	<LOQ = 0.1	<LOQ = 0.005
	Camomile *Matricaria chamomilla* L.	4	0.198–0.244 0.217	0.587–2.99 1. 572	<LOQ = 0.1	<LOQ = 0.005
	Common sunflower *Helianthus annuus* L.	3	<LOQ = 0.02	0.025–0.053 0.034	<LOQ = 0.1	<LOQ = 0.005
	Small-leaved lime *Tilia cordata* Mill.	3	0.057–0.077 0.047	0.119–0.284 0.207	<LOQ = 0.1	<LOQ = 0.005
	White nettle *Lamium album* L.	3	<LOQ = 0.02	0.426–0.585 0.499	<LOQ = 0.1	< LOQ = 0.005
Fruits	Dog rose *Rosa canina* L.	3	<LOQ = 0.02	<LOQ = 0.01	<LOQ = 0.1	<LOQ = 0.005
	Rowan *Sorbus aucuparia* L.	4	0.030–0.052 0.041	0.048–0.062 0.056	<LOQ = 0.1	<LOQ = 0.005
	Midland hawthorn *Crataegus oxyacantha* L.	3	<LOQ = 0.02–0.520 0.322	0.034–0.295 0.175	<LOQ = 0.1	<LOQ = 0.005
	Chasteberry *Vitex agnus-castus* L.	3	<LOQ = 0.02	<LOQ = 0.01	<LOQ = 0.1	<LOQ = 0.005
Seeds	Common flax *Linum usitatissimum* L.	24	<LOQ = 0.02–0.322 0.137	<LOQ = 0.01–0.707 0.089	<LOQ = 0.1	<LOQ = 0.005
	Narrowleaf plantain *Plantago lanceolata* L.	3	<LOQ = 0.02	0.025–0.053 0.034	<LOQ = 0.1	<LOQ = 0.005
Spices						
Herbs	Basil *Ocimum basilicum* L.	3	<LOQ = 0.02	1.34–1.92 1.66	<LOQ = 0.1	<LOQ = 0.005
Tubers	Common onion *Allium cepa* L.	3	0.036–0.060 0.047	0.126–0.305 0.231	<LOQ = 0.1	<LOQ = 0.005
	Common garlic *Allium sativum* L.	3	0.029–0.045 0.038	0.069–0.092 0.082	<LOQ = 0.1	<LOQ = 0.005
Rhizome	Turmeric *Curcuma longa* L.	3	<LOQ = 0.02	<LOQ = 0.01–0.256 0.138	<LOQ = 0.1	<LOQ = 0.005
Fruits	Black pepper *Piper nigrum* L.	26	<LOQ = 0.02–0.080 0.006	<LOQ = 0.01–0.740 0.159	<LOQ = 0.1–0.863 0.053	<LOQ = 0.005
	Peppers *Capsicum annuum* L.	8	<LOQ = 0.02–0.044 0.002	0.070–0.775 0.338	<LOQ = 0.01–0.178 0.077	<LOQ = 0.005
	Allspice *Pimenta dioica* L.	3	<LOQ = 0.02	0.309–0.422 0.361	<LOQ = 0.1	<LOQ = 0.005
Seeds	Cumin *Cuminum cyminum* L.	3	0.057–0.082 0.072	0.32–0.55 0.43	<LOQ = 0.1	<LOQ = 0.005
	Fennel *Foeniculum vulgare* Mill.	3	<LOQ = 0.02	0.253–0.382 0.321	<LOQ = 0.1	<LOQ = 0.005
	Fenugreek *Trigonella foenum-graecum* L.	3	0.026–0.041 0.034	0.159–0.202 0.173	<LOQ = 0.1	<LOQ = 0.005
Flowers	Clove tree *Eugenia caryophyllus* L.	3	<LOQ = 0.02	0.254–0.422 0.343	<LOQ = 0.1	<LOQ = 0.005
Tea and Coffee						
	Leaf of China tea	8	0.069–0.098 0.034	<LOQ = 0.01–0.150 0.070	<LOQ = 0.1	<LOQ = 0.005–0.007 0.006
	Beans of Arabica coffee	8	<LOQ = 0.02–0.088 0.058	0.021–0.791 0.140	<LOQ = 0.1	<LOQ = 0.005–0.007 0.006

**Table 3:** The health risk assessment related with the consumption of investigated products.

	Pb	Hg	Cd	As
* THQ				
Spices (mean)	1.07 × 10^−3^	0	1.80 × 10^−3^	4.33 × 10^−3^
Spices (max)	5.33 × 10^−3^	0	8.20 × 10^−3^	2.88 × 10^−2^
Herbs (mean)	1.41 × 10^−3^	1.00 × 10^−4^	1.94 × 10^−2^	2.70 × 10^−4^
Herbs (max)	1.58 × 10^−2^	6.70 × 10^−4^	2.17 × 10^−1^	1.78 × 10^−2^
Tea (mean)	7.50 × 10^−4^	7.70 × 10^−4^	1.31 × 10^−2^	0
Tea (max)	1.61 × 10^−3^	9.00 × 10^−4^	3.78 × 10^−2^	0
Coffee (mean)	4.33 × 10^−3^	1.11 × 10^−3^	6.46 × 10^−2^	0
Coffee (max)	2.45 × 10^−2^	2.60 × 10^−3^	9.81 × 10^−2^	0
** TTHQ				
Spices (mean)	7.20 × 10^−3^			
Spices (max)	4.23 × 10^−2^			
Herbs (mean)	2.12 × 10^−2^			
Herbs (max)	2.51 × 10^−1^			
Tea (mean)	1.46 × 10^−2^			
Tea (max)	4.03 × 10^−2^			
Coffee (mean)	7.01 × 10^−2^			
Coffee (max)	1.25 × 10^−1^			
*** EDI				
Spices (mean)	3.85 × 10^−3^	0	1.80 × 10^−4^	1.30 × 10^−3^
Spices (max)	1.92 × 10^−2^	0	8.20 × 10^−4^	8.63 × 10^−3^
Herbs (mean)	5.08 × 10^−3^	3.00 × 10^−5^	1.94 × 10^−3^	8.00 × 10^−5^
Herbs (max)	5.68 × 10^−2^	2.00 × 10^−4^	2.17 × 10^−2^	5.35 × 10^−3^
Tea (mean)	2.70 × 10^−3^	2.30 × 10^−4^	1.31 × 10^−3^	0
Tea (max)	5.79 × 10^−3^	2.70 × 10^−4^	3.78 × 10^−3^	0
Coffee (mean)	1.56 × 10^−2^	3.30 × 10^−4^	6.46 × 10^−3^	0
Coffee (max)	8.81 × 10^−2^	7.80 × 10^−4^	9.81 × 10^−3^	0

* THQ—target hazard quotient; ** TTHQ—total target hazard quotient; *** EDI—estimated daily intake.

**Table 4:** The carcinogenic risk (CR) assessment related with the consumption of investigated products.

	CR
Spices (mean)	1.95 × 10^−6^
Spices (max)	1.29 × 10^−5^
Herbs (mean)	1.20 × 10^−7^
Herbs (max)	8.03 × 10^−6^
Tea (mean)	0
Tea (max)	0
Coffee (mean)	0
Coffee (max)	0

## Data Availability

Raw data are available on request addressed to corresponding author.
